# Cancer-derived exosomal *Alu* RNA promotes colorectal cancer progression

**DOI:** 10.1038/s12276-024-01166-6

**Published:** 2024-03-14

**Authors:** Sara Magliacane Trotta, Antonio Adinolfi, Luca D’Orsi, Sonia Panico, Grazia Mercadante, Patrick Mehlen, Jayakrishna Ambati, Sandro De Falco, Valeria Tarallo

**Affiliations:** 1 Angiogenesis Lab, Institute of Genetics and Biophysics ‘Adriano Buzzati-Traverso’ - CNR, 80131 Naples, Italy; 2 BIOVIIIx srl, Via Alessandro Manzoni 1, 80123 Napoli, Italy; 3 Apoptosis, Cancer and Development Laboratory, Equipe labellisée ‘La Ligue’, LabEx DEVweCAN, Centre de Recherche en Cancérologie de Lyon, INSERM U1052-CNRS UMR5286, Université de Lyon, Centre Léon Bérard, 69008 Lyon, France; 4 Center for Advanced Vision Science; Department of Ophthalmology; Department of Pathology; Department of Microbiology, Immunology, and Cancer Biology, University of Virginia School of Medicine, Charlottesville, VA 22908 USA

**Keywords:** Colon cancer, Tumour biomarkers

## Abstract

Inflammation plays a crucial role in cancer progression, but the relevance of the inflammasome remains unclear. *Alu* RNA was the first endogenous nucleic acid shown to activate the NLRP3 (nucleotide-binding domain leucine-rich repeat containing 3) inflammasome. Here, we showed that *Alu* RNA can induce epithelial-to-mesenchymal transition (EMT) through NLRP3 inflammasome activation and IL-1β release in colorectal cancer (CRC) cells. *Alu* RNA is stored, transported and transferred to CRC cells by exosomes. Exosomal *Alu* RNA promotes tumorigenesis by inducing invasion, metastasis and EMT via NLRP3 inflammasome activation. Consistent with these data, we found that significantly increased *Alu* RNA expression correlates with the induction of NLRP3 priming in human CRC patients. Furthermore, the level of *Alu* RNA in circulating exosomes correlates with CRC progression in a preclinical model. These findings reveal the direct involvement of *Alu* RNA in cancer pathogenesis, and its presence in CRC cell-derived exosomes could be used as a noninvasive diagnostic biomarker.

## Exosomal Alu RNA: The new biomarker for non-invasive CRC diagnosis

*Alu* elements, parts of our DNA once considered useless, have been found to contribute to various diseases, including cancer. Researchers have been studying how these elements, especially free *Alu* RNAs, contribute to diseases like cancer. A recent study led by Dr. Valeria Tarallo focused on the impact of *Alu* RNAs on colorectal cancer. The research involved experiments on cancer cell lines (cells grown in a lab for study) and analysis of human tissue samples. The study aimed to explore how *Alu* RNAs cause changes in cells that lead to cancer progression. The researchers demonstrated that cancer cells released *Alu* RNAs via exosomes, which are extracellular vesicles involved in intercellular communication, and this release promotes tumorigenesis. The findings suggest that *Alu* RNAs could be used as biomarkers for CRC diagnosis and might offer new targets for therapy.

This summary was initially drafted using artificial intelligence, then revised and fact-checked by the author.

## Introduction

*Alu* elements are among the most abundant interspersed repetitive elements in the human genome, comprising almost 11% of nuclear DNA^1^, but at the time of their discovery, they were considered junk DNA. Typical *Alu* elements are approximately 300 nucleotides in length and do not encode any proteins. However, *Alu* elements can be transcribed either by RNA Pol II as a part of other transcripts (known as embedded *Alu* RNA) or by RNA Pol III (known as free *Alu* RNA). The role of embedded *Alu* sequences has been widely studied since they can cause human disorders through insertional mutagenesis of several genes; however, the role of free *Alu* RNA is less well known^2^. Free *Alu* RNAs are expressed at very low levels but can accumulate in response to cellular stress and malignant transformation, indicating that they could play crucial roles in pathological conditions^3,4^.

In 2011, we demonstrated that free *Alu* RNAs can cause a human disorder, namely, geographic atrophy, which is an advanced and untreatable form of age-related macular degeneration that causes blindness in millions of individuals^5^. The accumulation of *Alu* RNA in the retinal pigment epithelium (RPE) of human eyes causes cell death and degeneration through the activation of the nucleotide-binding domain leucine-rich repeat containing 3 (NLRP3) inflammasome. This innate immune complex senses pattern/danger-associated molecular patterns, resulting in interleukin-18 production and consequent RPE cell degeneration^6^. Since this discovery, specific roles of *Alu* elements have been described in other human disorders, such as systemic lupus erythematosus^7^, type II diabetes^8^ and Alzheimer’s disease^9^; however, their roles in cancer have remained the subject of debate. It has been suggested that *Alu* elements exert antitumor effects since they induce cytotoxicity in several cancer cell lines^10,11^. Furthermore, a more recent report suggested that they can also confer cancer resistance^12^. Despite these findings, hypomethylation of *Alu* elements has been reported in several cancers, and free *Alu* RNAs have been found to accumulate in hepatocellular carcinoma^13^. Moreover, we recently demonstrated that *Alu* expression is correlated with tumor growth and metastasis in human colorectal cancer (CRC) patients and triggers epithelial-to-mesenchymal transition (EMT) in several cancer cell lines^14^; these results suggest that *Alu* transcripts play a protumoral role in cancer. Consistent with these reports, tumor macrovesicles that were isolated from cancer patients have been shown to be markedly enriched in *Alu* transcripts^15^.

*Alu* RNA was the first endogenous nucleic acid shown to activate the NLRP3 inflammasome, and through this mechanism, *Alu* RNA induces RPE cell death^6^. *Alu* RNA has also been shown to induce inflammasome activation in other cell types, such as human monocytic leukemia cells (THP-1) and human cervical cancer cells (HeLa)^6^, suggesting that the inflammasome machinery can be activated by *Alu* RNA in cancer cells. The role of inflammasome activation in cancer is controversial; inflammasome activation has been described to either positively affect cell-autonomous death pathways and anticancer surveillance or to stimulate autocrine and paracrine processes that promote tumor growth and metastasis^16^. The baseline level of NLRP3 expression is not typically sufficient for NLRP3 inflammasome activation. Therefore, a two-step process that includes priming and activation is needed. Priming refers to increased transcription of *NLRP3* as well as the pro-forms of the inflammatory cytokines *IL1B* and *IL18*. Subsequently, the activation step induces the autoproteolysis of pro-caspase-1 into its cleaved and active fragment^17^. Although NLRP3 has been shown to be associated with EMT in CRC patients^18,19^ and several reports have correlated the secretion of IL-1β with EMT, tumor progression and metastasis^20–22^, the underlying molecular mechanism in CRC remains unknown.

Here, we demonstrated that *Alu* RNA induces EMT in CRC cell lines by activating the NLRP3 inflammasome and releasing IL-1β. We also showed that *Alu* RNA is stored and transported by exosomes and that CRC-derived exosomal *Alu* RNA can be transferred to cancer cells, in which it promotes cancer progression by inducing EMT via NLRP3 inflammasome activation. Finally, our preclinical data suggest that *Alu* RNA from circulating exosomes could be used as a biomarker for CRC diagnosis.

## Materials and Methods

### Cell culture

All the cancer cell lines were purchased from ATCC and cultured at 37 °C in 5% CO_2_. Human colon cancer cell lines (HCT116 and HT29) were grown in McCoy’s 5 A medium. Human colon cancer (SW480 and LS174T), human breast cancer (MCF7) and human cervix cancer (HeLa) cell lines were grown in Dulbecco’s modified Eagle’s medium. A human kidney carcinoma cell line (A498) was grown in Eagle’s minimum essential medium. A human lung cancer cell line (H460) was grown in RPMI-1640 medium. The media were supplemented with 10% heat-inactivated fetal bovine serum (FBS), 2 mM glutamine and a standard concentration of antibiotics (EuroClone). For OLT1177 dapansutrile (Merck, Sigma‒Aldrich) treatment, the cells were treated with 100 µM OLT1177 dapansutrile in complete medium. For anti-IL-1β (R&D) treatment, the cells were treated with 500 ng/ml anti-IL-1β in complete medium; an IgG isotype was used as a control.

### Human tissues

Snap-frozen tissue samples of primary human colorectal cancer, colorectal cancer liver metastases and corresponding adjacent normal tissues were obtained from the Biological Resources Center (CRB, Centre de Resource Biologique) of Centre Léon Bérard (protocol number: BB-0033-00050) (Lyon, France). The protocol involving the use of human material was approved by the CRB medical and scientific committee. All the patients provided informed consent to participate in the research according to French laws. RNA was extracted from human tissues with a QIACUBE kit (Qiagen) following the manufacturer’s protocol. The RNA concentration was measured with a Nanodrop system (Thermo Scientific), and the RNA quality was evaluated with a TapeStation system (Life Technologies).

### In vitro transcription of *Alu* RNA

We synthesized a 281-nucleotide *Alu* RNA sequence originating from the cDNA clone TS 103, which is known to be expressed in human cells (Shaik et al., 1997). The pT7/*Alu* plasmid was linearized with DraI and subjected to HiScribe T7 Quick High Yield RNA Synthesis Kit (BioLabs) according to the manufacturer’s instructions. After the T7 transcription reaction, the RNA was treated with DNase and purified by phenol–chloroform extraction and ethanol precipitation. RNA concentration was quantified via Nanodrop, and RNA integrity was assessed via gel electrophoresis. This process yields single-stranded RNA that folds into a defined secondary structure that is identical to that of Pol III-derived transcripts.

### Transient transfection

Cells were transfected with *Alu* RNA, *siNLRP3* (5’-GUUUGACUAUCUGUUCUdTdT-3’) or *siLuc* (5’-UAAGGCUAUGAAGAGAUdTdT-3’) (control for *siNLRP3*) using Lipofectamine 2000 (Invitrogen) according to the manufacturer’s instructions.

### Cell viability

Cell viability was assessed using the CellTiter 96 Aqueous One Solution Cell Proliferation Assay or Cell Titer-Glo® Luminiscent assay (Promega) according to the manufacturer’s instructions.

### ELISA

IL-1β levels that were secreted into conditioned cell culture media were analyzed using the Human IL-1β ELISA Kit (Abcam) according to the manufacturer’s instructions.

### Caspase-1 activation assay

Caspase-1 activity was measured using the Cell Meter Live Cell Caspase-1 Binding Assay Kit - Green Fluorescence - (AAT Bioquest, Inc.) according to the manufacturer’s instructions.

### Exosome purification, characterization and treatment

Exosomes were purified from CRC-derived conditioned media or serum samples from mice bearing CRC xenografts by ultracentrifugation. After transfection, the CRC cells were cultured in Dulbecco’s modified Eagle’s medium supplemented with exosome-depleted 5% fetal bovine serum (FBS). FBS was depleted of bovine exosomes by ultracentrifugation at 100,000 × *g* for 70 minutes. After 72 hours of incubation, the conditioned media were collected and centrifuged at 2,000 × *g* for 30 minutes to eliminate cells and debris. The supernatants were ultracentrifuged with an SW41Ti rotor at 14,000 × *g* for 30 minutes and then at 100,000 × *g* for 70 minutes. Exosomes were washed with phosphate-buffered saline (PBS) and collected by ultracentrifugation at 100,000 × *g* for 70 minutes. For transmission electron microscopy (TEM), the exosomes were fixed in 2% glutaraldehyde. The samples were applied to form var/carbon 100-mesh grids and incubated for 10 min. The grids were washed twice with filtered distilled water and stained with 1.5% UA in water for 10 min. Afterward, the cells were washed with water to remove the excess staining solution, the grids were air-dried. Images of the grids were acquired using an FEI Tecnai 12 transmission electron microscope (FEI Company, Hillsboro, Oregon, USA) equipped with a Veleta CCD digital camera (Olympus Soft Imaging Solutions GmbH, Münster, Germany) at 120 kV. For exosome-tracking experiments, purified exosomes were labeled using a PKH67 Green Fluorescent Cell Linker Mini Kit for Membrane Dye (Merck, Sigma‒Aldrich) according to the manufacturer’s instructions. Exosomes were purified from serum of a single mouse using a Total Exosomes Isolation Kit (from plasma) (Invitrogen) according to the manufacturer’s instructions.

### Western blotting analysis

The cell lines were lysed with lysis buffer (20 mM Tris-HCl, pH 8; 150 mM NaCl; 1% Triton X-100; 10 mM EDTA; 10% glycerol; and 1 mM ZnAc). The proteins were separated via SDS‒polyacrylamide gel electrophoresis, transferred to polyvinylidene difluoride membranes (Amersham Biosciences) and probed with the antibodies against the following proteins: Fibronectin (Merck, Sigma‒Aldrich, 1:1,000), Vimentin (Cell Signaling, 1:1,000), ZEB1 (Atlas Laboratories, 1:1,000), Cleaved Caspase-3 (Cell Signaling, 1:1000), Cleaved Caspase-1 (p20 fragment, AdipoGen, 1:1,000), CD63 (Thermo Scientific, 1:500), and E-Cadherin (mouse polyclonal, Cell Signaling Technology, 1:1,000). For normalization, we used an antibody against β-Tubulin (Elabioscience, 1:2000) or Vinculin (Cell Signaling Technology, 1:10,000). The secondary antibodies, namely, goat anti-rabbit (GeneTex) or anti-mouse HRP (ImmunoReagents), were diluted 1:10,000. The signals were visualized by chemiluminescence using an ECL substrate (Advansta) or by additional sensitive chemiluminescence using a LiteAblot Turbo (EuroClone) following the manufacturer’s instructions.

### Quantitative reverse transcription PCR

Total RNA was extracted from cells and exosomes using TRIzol reagent (Invitrogen) according to the manufacturer’s recommendations. Total RNA was reverse transcribed using a QuantiTec Reverse Transcription Kit (Qiagen). The RT products (cDNA) were amplified via real-time quantitative PCR (Applied Biosystems 7900 HT Fast Real-Time PCR system) with Power SYBR Green Master Mix. Oligonucleotide primers specific for human 18 S rRNA (forward 5′-CGCAGCTAGGAATAATGGAATAGG-3′ and reverse 5′-GCCTCAGTTCCGAAAACCAA-3′); *Alu* (forward 5′- CAACATAGTGAAACCCCGTCTCT-3′ and reverse 5′-TAGCTGGGACTACAGGCG-3′); human *NLRP3* (forward 5’-GCACCTGTTGTGCAATCTGAA-3’ and reverse 5’-TCCTGACAACATGCTGATGTGA-3’); human *IL-1β* (forward 5’-TTAAAGCCCGCCTGACAGA-3’ and reverse 5’-GCGAATGACAGAGGGTTTCTTAG-3’); human *IL-18* (forward 5’-ATCACTTGCACTCCGGAGGTA-3’ and reverse 5’-AGAGCGCAATGGTGCAATC-3’); and human *β-Actin* (forward 5′-CTCTTCCAGCCTTCCTTCCT-3′ and reverse 5′-TGTTGGCGTACAGGTCTTTG-3′) were used. The qPCR cycling conditions were 50 °C for 2 min and 95 °C for 10 min, followed by 40 cycles of a two-step amplification program (95 °C for 15 s and 58 °C for 1 min). At the end of the amplification, melting curve analysis was performed using the dissociation protocol from the Sequence Detection system to exclude contamination with nonspecific PCR products. The PCR products were also confirmed by agarose gel electrophoresis, which revealed only one specific band of the predicted size. For negative controls, no RT products were used as templates in the qPCR, and the results were verified by the absence of bands in the gel. The relative expression of the target genes was determined by the 2^–ΔΔCt^ method.

### Northern blotting

Total RNA (100-200 ng) was denatured for 10 minutes at 70 °C in Tris-borate-EDTA buffer–urea 2X loading buffer (Bio-Rad) and then run on a 10% denaturing Tris-borate-EDTA buffer–urea–polyacrylamide gel. Then, the RNA was transferred to positively charged nylon membranes (Amersham). The membranes were cross-linked by UV irradiation and saturated with a prehybridization solution (50% formamide, 0.12 M sodium phosphate buffer (pH 7.2), 0.25 M NaCl, 1 mM EDTA, and 7% SDS) at 42 °C for 1 hour. Then, 150 ng of denatured *Alu* probe was added and incubated for 16 hours at 42 °C. The *Alu* probe was obtained by PCR using the p*Alu* plasmid as the template and specific primers (*Alu* probe F: 5′-GGGCCGGGCGCGGTG-3′ and *Alu* probe R 5′GTACCTTTAAAGAGACAGAGTCTCGC-3′, biotinylated on the 5′-side); the following cycle parameters were used: 5 min at 95 °C, 1 min at 95 °C, 30 s at 58 °C, 1 min at 72 °C for 40 cycles and 8 min at 72 °C. The reaction products were then purified using ProbeQuant G-50 Micro Columns (GE Healthcare) following the manufacturer’s instructions. For normalization, we used a 5′-biotinylated probe against *5* *S* RNA (5′-AGCCTACAGCACCCGGTATT-3′) at a final concentration of 1 ng/μl. The membrane was washed twice with 2X SSC and 0.2% SDS at 65 °C. For detection, we used the Chemiluminescent Nucleic Acid Detection Module (Thermo Scientific) following the manufacturer’s instructions.

### Transwell invasion assay

Exosome-treated CRC cells (1 × 10^5^) were seeded into the upper chamber of a 24-well Transwell insert system with a polycarbonate filter with 8-μm pores (Corning) that was coated with 200 μg/ml Matrigel. The lower chamber was filled with complete medium as the positive control and with serum-free medium as the negative control. After 24 hours, the cells on the top of the filter were removed, and those on the bottom side were stained with 4’,6-diamidino-2-phenylindole (DAPI). Images were captured on a Nikon Eclipse Ni-E (Nikon, Corporation Tokyo, Japan) fluorescence microscope. For each group, seven random Transwell chamber fields were counted. Single cells were counted using ImageJ (NIH, Bethesda, MD, USA).

### Soft agar assay

Six-well plates were precoated with a 0.75% basal agar layer containing culture media. Exosome-treated CRC cells (1 × 10^5^) were resuspended in a 0.32% upper agar layer and seeded at a density of 1.25 × 10^4^ cells per well. The medium was changed every 3 days. After 15 or 21 days, cell colonies were fixed with 4% PFA and visualized by 0.005% crystal violet (Merck, Sigma‒Aldrich) staining. Images were captured, and the number of colonies was counted by ImageJ software (NIH, Bethesda, MD, USA).

### Colony formation assay

Exosome-treated CRC cells were plated at 500 cells/well in 6-well plates. After 7 days, the cell colonies were visualized by 0.005% crystal violet (Merck, Sigma‒Aldrich) staining. Images were captured, and the number of colonies was counted by ImageJ software (NIH).

### Animals

CD1 mice were purchased from Charles River. The animal experiments were conducted in accordance with European directives no. 2010/63/UE and Italian directives D.L. 26/2014 and were approved by the Italian Ministry of Health (Authorization no. 492/2020-PR of 19/05/2020). For all the procedures, 7- to 8-week-old male mice were used, and anesthesia was achieved by intraperitoneal injection of 100 mg/kg ketamine hydrochloride and 10 mg/kg xylazine.

### Xenograft tumor model

A total of 3 × 10^6^ HCT116 cells were subcutaneously injected into the right flanks of CD1 nude mice. Tumor volume (mm^3^) was quantified three times a week by measuring the shortest (d) and longest (D) tumor diameters with an electronic caliper, the tumor volume was calculated using the formula D × d^2^/2. Seventeen days after injection, the mice were sacrificed, the serum was collected to purify exosomes, and the tumor masses were harvested and weighed. For the tail vein metastasis assay, 1 × 10^6^ HCT116 cells were treated with Exo Mock or Exo *Alu* RNA for 24 hours and then intravenously injected into CD1 nude mice (*n* = 8 mice per group) via the tail vein. Four weeks after the injection, the lungs were explanted, and the sera were collected to purify exosomes.

### Immunohistochemical staining

Mouse lungs were fixed in 4% PFA and embedded in paraffin. Immunostaining was performed on 7-μm-thick sections. Antigen retrieval was performed for 15 minutes at 37 °C in 0.2% trypsin and 0.001% CaCl_2_ solution. Endogenous peroxidase activity was blocked with 0.3% H_2_O_2_ for 30 minutes. After blocking with 10% goat serum and 1% BSA in PBS/0.1% Triton-X-100, the slides were stained with the primary Anti-Human Nuclei Antibody clone 3E1.3 (Merck, Sigma‒Aldrich, 1:200) or anti-Cleaved Caspase-1 (p20 fragment; AdipoGen, 1:100) for 16 hours at 4 °C. Then, the slides were stained with goat anti-mouse biotinylated secondary antibodies (DAKO), and signal amplification was performed using a Vectastain elite ABC kit (Vector Laboratories). The signal was visualized using an ultraView Universal DAB Detection Kit (Merck, Sigma‒Aldrich). The slides were counterstained with hematoxylin. All counts were performed on five different random fields from at least four random sections for each animal. All the images were recorded with a Leica DC480 digital camera (Leica).

### Statistical analysis

The data are expressed as the mean ± SEM, with *p* < 0.05 considered to indicate statistical significance. Differences among groups were tested by one-way ANOVA.

## Results

### *Alu* RNA induces EMT through NLRP3 inflammasome activation in CRC cell lines

**Fig. 1:**
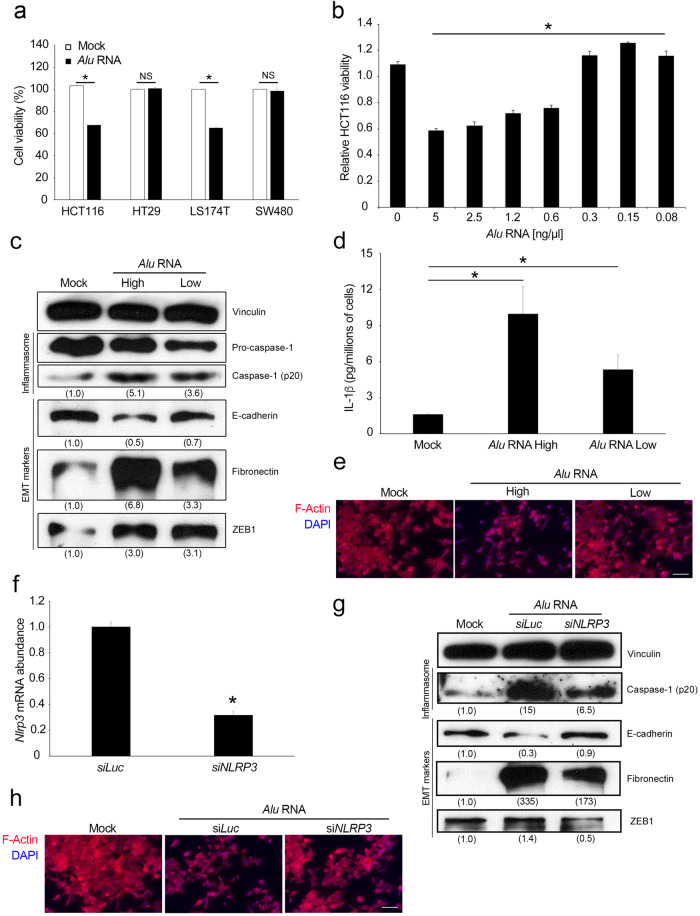
*Alu* RNA induces EMT through NLRP3 inflammasome activation in CRC cell lines. **a** *Alu* RNA exerts cytotoxic effects on HCT116 and LS174T cells but does not alter the viability of HT29 and SW480 cells, as determined by the MTT assay (*n* = 3). **b** Dose-dependent effects of *Alu* RNA on HCT116 cell viability, as determined by the MTT assay (*n* = 3). **c** Western blotting analysis showing the activation of Caspase-1, increased expression of the mesenchymal marker Fibronectin, decreased expression of E-cadherin and increased expression in the EMT transcription factor ZEB1 in HCT116 cells after transfection with high or low levels of *Alu* RNA. Densitometric values are normalized to Vinculin and are shown in parentheses. **d** High and low *Alu* RNA levels induce the secretion of IL-1β from HCT116 cells, as measured by ELISA. IL-1β protein levels were normalized to the number of adherent cells (*n* = 3). **e** Representative images of HCT116 cells transfected with vehicle (Mock, left), high *Alu* RNA levels (middle), or low *Alu* RNA levels (right) and stained for F-actin with rhodamine phalloidin (red). Nuclei were counterstained with 4’,6-diamidino-2-phenylindole (DAPI, blue). Scale bar: 100 μm. **f** Transfection with an siRNA targeting *NLRP3* (*siNLRP3*) reduced the abundance of the target mRNA in HCT116 cells compared with that in cells transfected with a control siRNA (si*Luc)*, as determined by qRT‒PCR (*n* = 3). *siNLRP3* transfection prevented *Alu*-induced EMT in HCT116 cells, as determined by (**g**) western blotting and **h** immunofluorescence analyses. *Alu* RNA (central lane, **g**) induces Caspase-1 activation as well as EMT, decreasing E-cadherin expression and increasing Fibronectin and ZEB1 expression compared to those in the Mock group (left lane, **g**). Transfection of *siNLPR3*-knockdown cells with *Alu* RNA prevented *Alu*-induced EMT and inhibited *Alu*-induced Caspase-1 activation (right lane, **g**). Densitometric values are normalized to Vinculin and are shown in parentheses. **h** Representative images of F-actin (red) staining. Nuclei were counterstained with DAPI (blue). Scale bar: 100 μm. For all panels, *p < 0.05; NS = not statistically significant. The error bars denote the s.e.m.

We previously demonstrated that *Alu* RNA induces EMT in SW480 colorectal cancer cells without affecting cell proliferation^14^. In contrast, other reports have shown that *Alu* RNA exerts cytotoxic effects on other cancer cell lines^6,10,11^. To better understand the role of *Alu* retroelements and the downstream pathways that are activated during CRC progression, we tested the effect of *Alu* RNA overexpression on different CRC cell lines by transfecting them with a synthetically transcribed RNA. As shown in Fig. 1a, *Alu* RNA exerted cytotoxic effects on two CRC cell lines (HCT116 and LS174T) as well as on other cancer cell lines, independent of their tissue of origin, such as kidney (A498), lung (H460), cervix (HeLa), and breast (MCF7) (Supplementary Fig. 1). Conversely, *Alu* RNA did not alter the viability of either the HT29 or SW480 CRC cell lines, which is consistent with our previous report^14^ (Fig. 1a). Consistent with these results, western blotting analysis revealed an increase in Caspase-3 cleavage in only *Alu*-sensitive (HCT116 and LS174T) but not in *Alu*-resistant (HT29 and SW480) CRC cell lines (Supplementary Fig. 2).

Our data support the idea that *Alu* RNAs promote CRC progression, although these sequences are cytotoxic to most cancer cell lines. Since *Alu* RNA is present at extremely low levels under pathophysiological conditions compared to those we previously observed in vitro, we decided to study the dose-dependent effects of *Alu* RNA transfection on HCT116 cells. Interestingly, we found that this mechanism is carefully regulated. Indeed, as shown in Fig. 1b, high levels of *Alu* RNA induced cytotoxicity in HCT116 cells, whereas low doses of *Alu* RNA, approximately thirty times lower than the amount we used for transfection, induced a slight but significant increase in HCT116 cell viability.

Since *Alu* RNA has been shown to activate the NLRP3 inflammasome^6^, we investigated whether this pathway is also activated in CRC cells. Interestingly, we found that both high and low levels of *Alu* RNA induce inflammasome priming in HCT116 cells by upregulating the mRNA expression of *NLRP3* and *IL-1B*, while no change in the expression of *IL18* was observed, as shown by qRT‒PCR (Supplementary Fig. 3). Additionally, both levels of *Alu* RNA activated the NLRP3 inflammasome, leading to Caspase-1 activation and the release of IL-1β, as measured by western blotting (Fig. 1c) and ELISA (Fig. 1d), respectively. Next, we investigated whether *Alu* RNA induces EMT in HCT116 cells, as we previously demonstrated in SW480 cells^14^. As shown in Fig. 1c, western blotting analysis revealed that both high and low levels of *Alu* RNA induce EMT in HCT116 cells, as shown by the downregulation of E-cadherin expression and upregulation of Fibronectin, Vimentin and ZEB1, which are EMT transcription factors whose expression is strongly induced by *Alu* RNA. Consistent with these findings, immunofluorescence analyses confirmed that both high and low levels of *Alu* RNA promoted cytoskeleton reorganization (Fig. 1e). Collectively, these data suggest that, even in low amounts, *Alu* RNA can activate the NLRP3 inflammasome and promote EMT in the HCT116 cell line.

Since IL-1β has been shown to induce EMT in cancer^20,21^, we hypothesized that *Alu* RNA could induce EMT through NLRP3 inflammasome activation. To test this hypothesis, we performed a rescue experiment in which the HCT116 cell line was transfected with a siRNA targeting *NLRP3* mRNA before *Alu* RNA was introduced. An siRNA against *Luciferase* mRNA was used as a control. As shown in Fig. 1f, si*NLRP3* efficiently reduced *NLRP3* expression in HCT116 cells, as shown by qRT‒PCR. Next, we monitored the expression levels of EMT markers via western blotting once inflammasome activation was impaired. Consistent with previous data, our results confirmed that, compared with the mock control, *Alu* RNA (central lane, Fig. 1g) induced Caspase-1 cleavage and EMT, reduced E-cadherin levels and increased the expression of mesenchymal markers (Fibronectin, Vimentin and ZEB1) compared to the mock condition (left lane, Fig. 1g). Surprisingly, the transfection of *Alu* RNA into si*NLRP3*-transfected cells reversed *Alu*-induced EMT by reducing Caspase-1 activation (right lane, Fig. 1g). F-actin staining confirmed that knockdown of *NLRP3* prevented the *Alu* RNA-induced acquisition of the mesenchymal phenotype (Fig. 1h). Consistent with these data, pharmacological inhibition of NLRP3 with dapansutrile (OLT1177)^23^ also inhibited *Alu* RNA-induced EMT as a consequence of impaired NLRP3 inflammasome activation (Supplementary Fig. 4). Furthermore, we also found that a blocking antibody against IL-1β was able to prevent *Alu* RNA-induced EMT, thus providing further evidence for the involvement of IL-1β in promoting *Alu* RNA-induced mesenchymal changes (Supplementary Fig. 5). Next, we investigated whether the NLRP3 inflammasome is also activated in SW480 cells. We demonstrated that *Alu* RNA induces EMT, but we were not able to detect either the cleavage of Caspase-1 or the secretion of interleukin-1β/18 by western blotting analysis^14^. Consistent with these results, we found that *Alu* RNA could prime the NLRP3 inflammasome (Supplementary Fig. 6a) and trigger a slight but significant increase in Caspase-1 activation, as shown with a fluorometric assay (Supplementary Fig. 6b); these findings further suggested that EMT occurs downstream of the inflammasome. Collectively, these data demonstrate that *Alu* RNA induces EMT through the activation of the NLRP3 inflammasome and the release of IL-1β in CRC cells.

### *Alu* RNA is released from CRC cells via exosomes

**Fig. 2:**
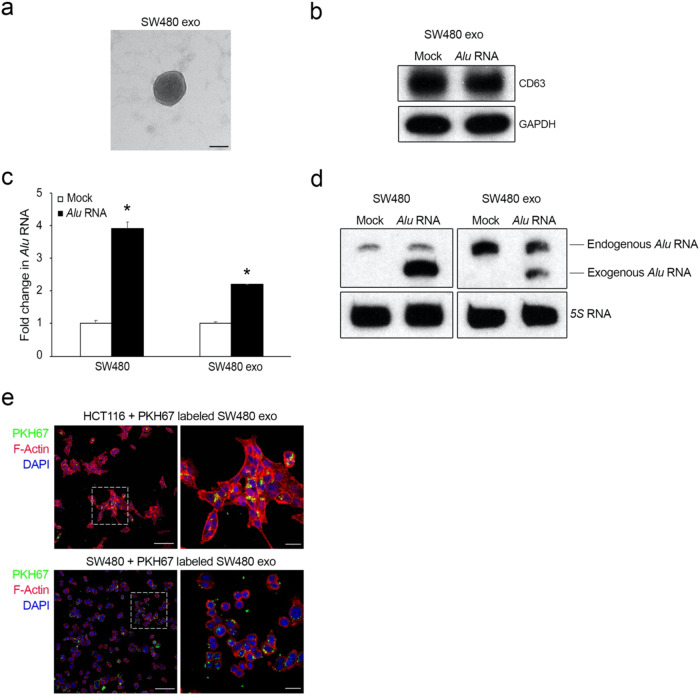
*Alu* RNA is stored and transported by exosomes by CRC cells. **a** TEM image showing vesicles with morphologies and sizes that are characteristic of exosomes. Scale bar: 50 nm. **b** Western blotting analysis of CD63 levels in exosomes that were purified by ultracentrifugation from the culture supernatants of SW480 cells (SW480 Exo) that were transfected with *Alu* RNA or vehicle (Mock). GAPDH was used as a loading control. **c** qRT‒PCR results showing the abundance of *Alu* RNA in exosomes derived from *Alu* RNA-transfected SW480 cells (SW480 Exo) compared to that in exosomes derived from vehicle-transfected cells (Mock). On the left, as a control, the same analysis was performed on *Alu* RNA- or vehicle (Mock)-transfected SW480 cells (*n* = 3); *p < 0.05. Error bars denote the s.e.m. **d** Northern blotting analysis shows the abundance of *Alu* RNA in exosomes from SW480 cells (SW480 Exo) transfected with *Alu* RNA compared to those from vehicle-transfected cells (Mock). On the left, as a control, the same analysis was performed on *Alu* RNA- or vehicle (Mock)-transfected SW480 cells. We observed a band corresponding to endogenous *Alu* RNA (approximately 300 nucleotides in length) and a faster band corresponding to the transfected exogenous *Alu* RNA (281 nucleotides in length). 5S RNA was used as a loading control (*n* = 3). **e** Representative confocal images of PKH67-labeled SW480-derived exosomes (green) in HCT116 and SW480 cells that were stained for F-actin with rhodamine phalloidin (red). Nuclei were counterstained with DAPI (blue). Scale bar: 100 μm. A higher magnification is shown on the right. Scale bar: 25 μm.

Retrotransposon elements, such as *Alu* sequences, are enriched in tumor-derived exosomes^15^; thus, we investigated whether *Alu* RNA could promote CRC progression via exosome transport. To address this question, in vitro transcribed *Alu* RNA was transfected into SW480 CRC cells, and exosomes were isolated from the culture supernatants of SW480/*Alu* RNA and control cells (SW480/Mock) by a combination of centrifugation and ultracentrifugation. The purified exosomes were identified and characterized by TEM, dynamic light scattering (DLS) and western blotting analysis. TEM revealed that exosomes that were purified from SW480 cell-conditioned media were membrane-encapsulated particles ranging from 100 to 130 nm in size (Fig. 2a). DLS analysis, the results of which are shown in Supplementary Fig. 7, revealed that the size distributions of exosomes that were released by SW480/*Alu* RNA and SW480/Mock cells were homogenous. Western blotting analysis revealed that the vesicles were positive for the exosome marker CD63 (Fig. 2b). Finally, we analyzed total RNA that was isolated from exosomes and measured the expression of *Alu* transcripts. qRT‒PCR analysis revealed significant accumulation of *Alu* transcripts in exosomes derived from SW480/*Alu* RNA cells compared to exosomes derived from control cells (Fig. 2c). This result was further confirmed by northern blotting analysis, where the presence of a faster band corresponding to exogenous and transfected *Alu* RNA was also clearly evident (Fig. 2d). As a control, the same analyses were carried out on nontransfected cells (Fig. 2c, d). Next, SW480 and HCT116 cells were incubated with PKH67-labeled exosomes that were derived from SW480 cells, and as shown in Fig. 2e, both CRC cell lines were able to internalize the exosomes. These results clearly demonstrated that Pol lII-derived *Alu* transcripts are stored and transported by CRC cells via exosomes.

### Cancer-derived exosomal *Alu* RNA promotes tumorigenesis

**Fig. 3:**
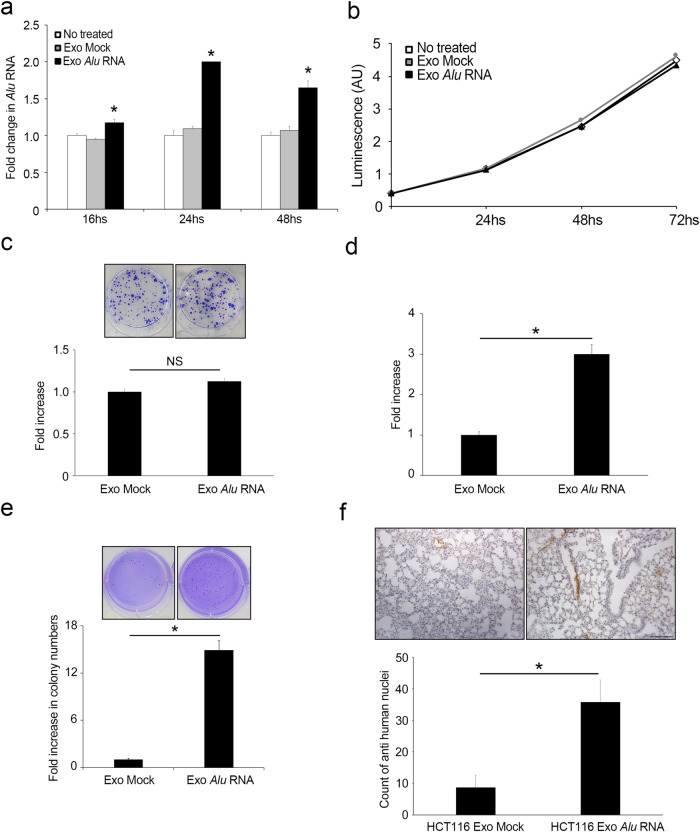
Cancer-derived exosomal *Alu* RNA promotes tumorigenesis and metastasis dissemination. **a** qRT‒PCR analysis of *Alu* RNA expression in HCT116 cells treated with exosomes from *Alu-*transfected SW480 cells (Exo *Alu*) after 16, 24 and 48 hours compared with that in HCT116 cells treated with control exosomes (Exo Mock) or untreated cells (*n* = 3); *p < 0.05. Error bars denote the s.e.m. **b** Exosomal *Alu* RNA did not alter HCT116 cell viability over time, as determined by luminescence assay (*n* = 3). **c** Exosomal *Alu* RNA does not alter the ability of HCT116 cells to form colonies. The data are expressed as the fold increase compared with HCT116 cells treated with Exo Mock (*n* = 3). Error bars denote the s.e.m. Representative images of the two conditions are shown. **d** Exosomal *Alu* RNA increases HCT116 cell invasion. The data are expressed as the fold increase compared with HCT116 cells treated with Exo Mock (*n* = 3); *p = 0.019. Error bars denote the s.e.m. **e** Exosomal *Alu* RNA increases the ability of HCT116 cells to grow in an anchorage-dependent manner. The bars indicate the average number of colonies in each well (*n* = 3); *p = 0.001. Error bars denote the s.e.m. Representative images of the two conditions are shown. **f** Exosomal *Alu* RNA increases the metastatic potential of HCT116 cells in vivo. The bars indicate the average number of human nuclei-positive cells in lung sections from mice that were injected with Exo *Alu*-pretreated HCT116 cells via the tail vein compared to that in lung sections from mice that were injected Exo Mock-pretreated HCT116 cells; *p = 0.012. Error bars denote the s.e.m. Representative images of anti-human nuclei-stained lung sections are shown. Scale bar: 100 μm.

We then investigated whether *Alu* RNA could be transferred via exosomes to HCT116 and SW480 cells. Exosomes derived from SW480/Mock (Exo Mock) and SW40/*Alu* RNA (Exo *Alu*) cells were cocultured with HCT116 cells at different time points. The qRT‒PCR analysis results in Fig. 3a show that compared to untreated cells and HCT116 cells that were incubated with Exo Mock, HCT116 cells that were incubated with Exo *Alu* exhibited an increase in *Alu* RNA after 16 hours of incubation, with a peak at 24 hours, and this increase was sustained at 48 hours. Similar expression kinetics were also observed when SW480 cell-derived exosomes were cocultured with SW480 cells (Supplementary Fig. 8); these results indicated that exosomal *Alu* RNA can be transferred to recipient cells.

Next, we investigated whether exosomal *Alu* RNA could affect CRC cell viability. Interestingly, we did not observe significant effects on HCT116 or SW480 cell growth, as shown in Fig. 3b and Supplementary Fig. 9a. Consistent with these findings, plate colony formation assays revealed no difference in growth between HCT116 and SW480 cells that were treated with Exo *Alu*) and cells that were treated with Exo Mock, as shown in Fig. 3c and Supplementary Fig. 9b. Interestingly, compared with Exo Mock, Exo *Alu* significantly promoted invasion and increased the ability of HCT116 and SW480 cells to grow in an anchorage-independent manner (Fig. 3d, e and Supplementary Fig. 10a, b, respectively), indicating that the transfer of *Alu* transcripts to CRC cells promotes a protumoral phenotype in in vitro cell-based assays.

Finally, we investigated the ability of exosomal *Alu* RNA to increase cell metastatic potential in vivo. HCT116 cells were cocultured with exosomes enriched with *Alu* RNA or control exomes for 24 hours before being intravenously injected into immunocompromised CD1 mice via the tail vein. After four weeks, the lungs were harvested, and the ability of the HCT116 cells to invade pulmonary tissues was evaluated via immunohistochemical analysis using an antibody that specifically recognizes human nuclei. Interestingly, we found that the lungs of mice that were injected with Exo *Alu*-pretreated cells contained significantly more human nuclei than the lungs of mice that were injected with control exosome-pretreated cells (Fig. 3f). Notably, all the lungs that were explanted from mice in the Exo *Alu* group were positive for human nuclei, while one-third of the lungs in the control group were not positive for human nuclei. This further indicated that exosomal *Alu* RNA increases the metastatic potential of HCT116 cells. Collectively, these data demonstrated that cancer-derived exosomal *Alu* RNA induces tumorigenesis and promotes metastasis dissemination.

### Exosomal *Alu* RNA induces tumorigenesis via NLRP3 inflammasome activation

**Fig. 4:**
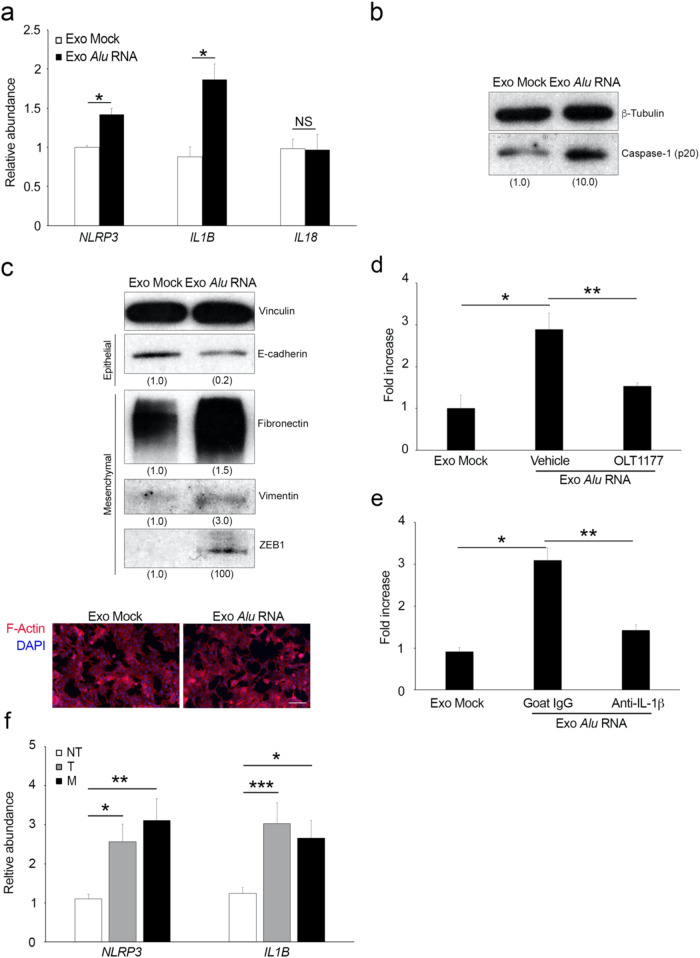
Exosomal *Alu* RNA induces tumorigenesis via NLRP3 inflammasome activation. **a** Exosomal *Alu* RNA induces NLRP3 inflammasome priming by increasing the mRNA expression of *NLRP3* and *IL-1B* in HCT116 cells, as shown by qRT‒PCR (*n* = 3); *p < 0.05. No difference in *IL18* gene expression was observed. The error bars denote the s.e.m. **b** Western blotting analysis showing the activation of Caspase-1 (p20) in HCT116 cells that were treated with Exo *Alu*. Densitometric values are normalized to β-Tubulin and are shown in parentheses. **c** Western blotting analysis (above) showing a decrease in E-cadherin expression, an increase in mesenchymal marker expression (Fibronectin and Vimentin), and an increase in EMT-TF ZEB1 expression in HCT116 cells treated with Exo *Alu*. Densitometric values are normalized to Vinculin and are shown in parentheses. Below, representative images of HCT116 cells that were treated with Exo Mock or Exo *Alu* and stained for F-actin (red). Nuclei were counterstained with DAPI (blue). Scale bar: 100 μm. **d** OLT1177 (100 μM) reduces exosomal *Alu* RNA-induced HCT116 cell invasion. The data are expressed as the fold increase compared with HCT116 cells treated with Exo Mock (*n* = 3); *p = 0.015; **p = 0.027. Error bars denote the s.e.m. **e** Anti-IL1β (500 ng/ml) reduces exosomal *Alu* RNA-induced HCT116 cell invasion. The data are expressed as the fold increase compared with HCT116 cells that were treated with Exo Mock (n = 3); *p = 0.026; **p = 0.047. Error bars denote the s.e.m. **f** *NLRP3* and *IL-1B* mRNA expression in 13 matched nontumoral (NT) tissues, primary colon tumor (T) tissues and liver metastasis (M) tissues from patients was evaluated by qRT‒PCR and normalized to *18* *S* RNA expression; *p = 0.03; **p = 0.014; ***p = 0.019. The error bars denote the s.e.m.

We then evaluated whether activation of the NLRP3 inflammasome and induction of EMT were involved in exosomal *Alu* RNA-mediated induction of tumorigenesis in CRC cells. Compared with control exosomes, exosomal *Alu* RNA induced inflammasome priming by upregulating *NLRP3* and *IL-1B* mRNA expression. These effects occurred to equivalent degrees in both HCT116 and SW480 CRC cells, as shown in Fig. 4a and Supplementary Fig. 11a, respectively. Consistent with this result, we observed Caspase-1 activation in HCT116 cells, as shown by western blotting analysis (Fig. 4b), and in SW480 cells, as shown by flow cytometry analysis (Supplementary Fig. 11b). Next, we assessed whether exosomal *Alu* RNA promoted EMT in these cells. As shown in Fig. 4c, we observed a decrease in the epithelial marker E-cadherin; an increase in the mesenchymal markers, Fibronectin and Vimentin; and an increase in the transcription factor ZEB1 in HCT116 cells that were treated with Exo *Alu* RNA, as shown by western blot. These changes were associated with the acquisition of a mesenchymal phenotype by HCT116 cells that were treated with Exo *Alu* RNA, as shown by immunofluorescence analysis of F-Actin. Similarly, exosomal *Alu* RNA induced a mesenchymal phenotype in SW480 cells, as shown by western blotting analysis (Supplementary Fig. 12). Consistent with previous data, we found that both dapansutrile (OLT1177) and a blocking antibody against IL-1β inhibited the ability of Exo *Alu* RNA to promote HCT116 cell invasion (Fig. 4d, e), suggesting that the NLRP3-IL1β pathway is crucial for the exosomal *Alu* RNA-mediated increase in HCT116 cell invasion. Consistent with these data, we found that lung micrometastases, which were observed only in mice that were injected with Exo *Alu-*pretreated HCT116 cells, were positive for Caspase-1 (Supplementary Fig. 13); these data demonstrated that the NLRP3 inflammasome was activated in these cells in vivo. Collectively, these data indicate that activation of the NLRP3 inflammasome is a critical mediator of the induction of tumorigenesis by exosomal *Alu* RNA.

Next, we investigated whether human CRC tumors with *Alu* RNA accumulation, which is associated with progression and malignancy^14^, also displayed evidence of inflammasome activation. *NLRP3* and *IL1B* mRNA expression was quantified in 13 matched nontumoral (NT) tissues, primary colon tumor (T) tissues and liver metastasis (M) tissues. Interestingly, we found that both *NLRP3* and *IL1B* mRNA levels were significantly upregulated in both primary colon tumor (T) and liver metastasis (M) tissues compared with nontumoral (NT) tissues (Fig. 4f). These data provide evidence that *Alu* RNA accumulation in human CRC tumors induces the priming of the inflammasome, which was consistent with the functional data from the cell culture studies.

### Exosomal *Alu* RNA abundance is correlated with CRC progression

**Fig. 5:**
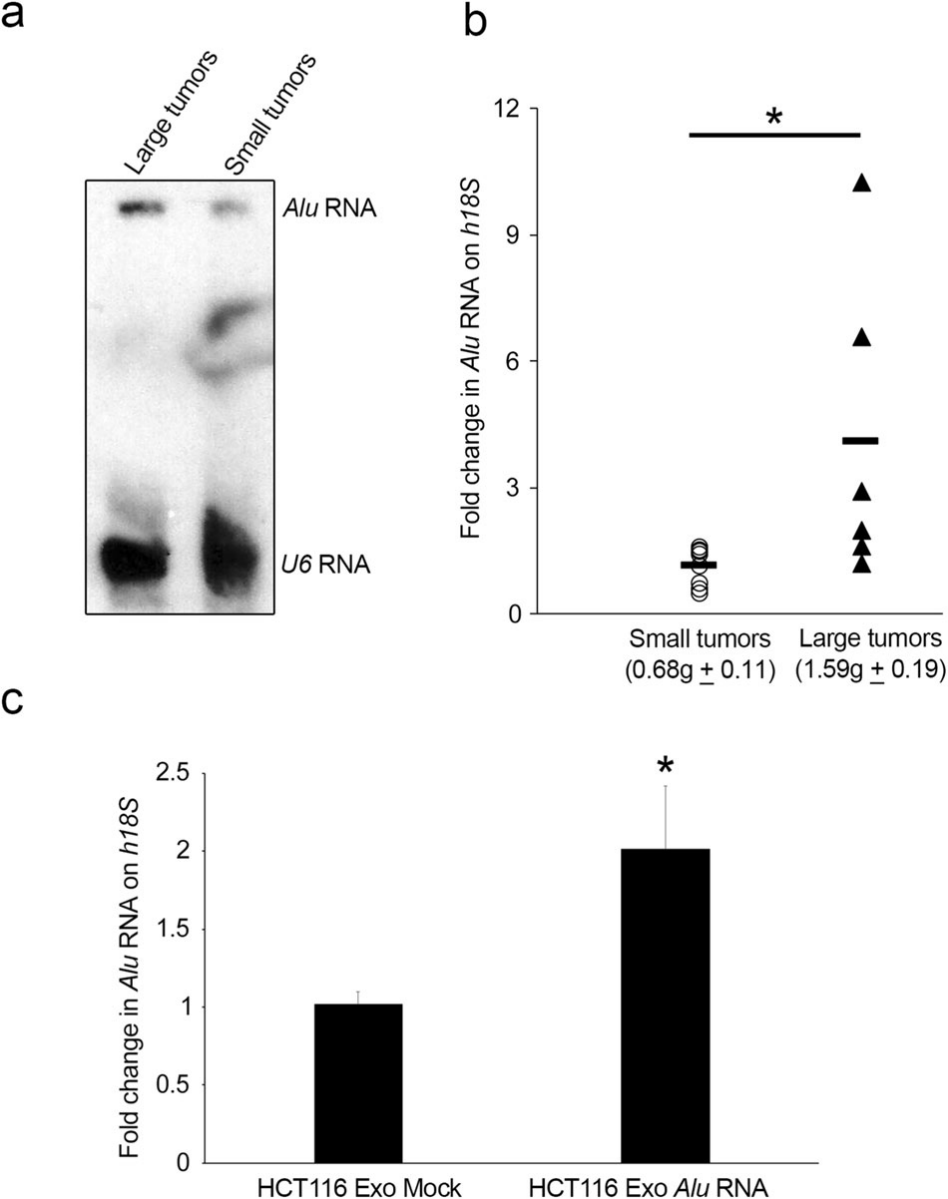
Exosomal *Alu* RNA abundance is correlated with CRC progression. **a** Northern blotting analysis showing the abundance of *Alu* RNA in exosomes that were purified by ultracentrifugation from serum of xenotransplanted mice bearing large (*n* = 8; 1.52 ± 0.09) or small CRC tumors (*n* = 6; 0.88 ± 0.14). *U6* RNA was used as a loading control. **b** qRT‒PCR analysis showing *Alu* RNA levels in exosomes purified from the serum of xenotransplanted mice bearing small (*n* = 8; 0.68 g±0.11) or large (*n* = 6; 1.58 g±0.19) CRC tumors, as evaluated by qRT‒PCR and normalized to human *18* *S* RNA; *p = 0.037. **c** qRT‒PCR analysis showing *Alu* RNA levels in exosomes that were purified from the serum of mice that were intravenously injected with Exo *Alu-* or Exo Mock-pretreated HCT116 cells. The values were normalized to human *18* *S* RNA; *p = 0.025. The error bars denote the s.e.m.

Since one of the major applications of exosomes is diagnosis, we investigated whether the abundance of *Alu* RNA could be used as a biomarker of cancer. Therefore, we analyzed whether the exosomal *Alu* RNA content was correlated with malignancy in vivo. We isolated RNA from exosomes that were purified from the serum of mice that were xenotransplanted with human colorectal cancer cells bearing large and small tumors. As shown by the northern blot in Fig. 5a, we observed an increase in the accumulation of *Alu* transcripts in exosomes from the serum of mice bearing large tumors compared to those from the serum of mice bearing small tumors. Moreover, these results were confirmed by a second independent experiment in which we analyzed the abundance of exosomal *Alu* RNA in each mouse by qRT‒PCR analysis. As shown in Fig. 5b, we observed a significant increase in the expression of exosomal *Alu* RNA in large tumors compared to that in small tumors, and *Alu* RNA expression normalized to human 18S rRNA expression. Furthermore, we analyzed the abundance of *Alu* RNA in exosomes from the serum of mice that had been intravenously injected with Exo *Alu* RNA-pretreated HCT116 cells (Fig. 3f) to assess whether exosomal *Alu* RNA contents correlate with metastasis in vivo. Interestingly, we observed a significant increase in *Alu* transcript levels in exosomes from mice that were injected with Exo *Alu* RNA-pretreated cells compared to those from mice that were injected with control cells (Fig. 5c).

Collectively, these data clearly show that the exosomal *Alu* RNA contents correlate with CRC progression in vivo and that these transcripts could be used as a biomarker of cancer.

## Discussion

Our data define the molecular mechanism by which Pol III-derived *Alu* transcripts promote the progression of CRC. We showed that cancer-derived exosomal *Alu* RNA can be delivered to CRC cells and promote cancer progression by inducing EMT through NLRP3 inflammasome activation. Furthermore, we found that *Alu* transcript abundance in exosomes is correlated with CRC progression in preclinical models. Overall, our study reveals the direct role of *Alu* RNA retroelements in cancer pathogenesis and their clinical significance.

We found that *Alu* RNA plays dual opposing roles in regulating CRC cell fate. Consistent with the findings of previous reports^5,10,11,14^, we found that the transfection of in vitro-transcribed *Alu* RNA can either cause cell death or exert no effect on cell viability. This variability could be attributed to differences in the concentration of accumulated *Alu* RNA as well as to differences in the mutational status of tumor suppressor genes in the cancer cell lines that were analyzed. Therefore, future studies should aim to better elucidate the molecular mechanisms underlying these differences. However, we observed similar downstream signal transduction in both *Alu*-sensitive and *Alu*-resistant CRC cell lines, even when the amount of transfected *Alu* RNA was extremely low.

Furthermore, both high and low levels of *Alu* RNA induce NLRP3 inflammasome activation in CRC cell lines, resulting in the secretion of IL-1β. A high level of *Alu* RNA leads to cell death, whereas a low level of *Alu* RNA slightly but significantly induces proliferation. IL-1β has been shown to be secreted upon cell death due to plasma membrane rupture^24^ or pyroptosis, which is a lytic form of cell death that is triggered by Caspase-1 activation^25^; however, a recent study demonstrated that cell death is not essential for Caspase-1-mediated IL-1β activation and secretion^26^, and this cell death-independent mechanism likely occurs in CRC cells.

However, further investigation is needed to elucidate the mechanism by which *Alu* RNA activates the NLRP3 inflammasome in CRC cell lines. Previous studies have shown that although no physical interaction occurs between *Alu* RNA and NLRP3, *Alu* RNA triggers NLRP3 activation in RPE cells through the generation of reactive oxygen species (ROS) and the activation of the ATP-gated P2X7 channel (P2X7R)^6,27^. We hypothesize that *Alu* RNA may activate the NLRP3 inflammasome in CRC cells through a similar pathway. However, it is important to consider that the context of CRC is likely more complex than that of RPE cells, as the transfection of *Alu* RNA into CRC cells can either induce cell death or exert no effect on cell viability.

Due to its broad activity in shaping the immune response, the role of the NLRP3 inflammasome in CRC development is complex and controversial; the NLRP3 inflammasome has been reported to exert both inhibitory and promoting effects on tumorigenesis. The role of the NLRP3 inflammasome in protecting against colitis-associated colon cancer has been well established, and it helps to resolve inflammation and prevent the development of cancer^28^. However, chronic inflammation can also promote carcinogenesis, and activation of the NLRP3 inflammasome has been associated with numerous human malignancies, including CRC. Genetic variants in the *NLRP3* gene have been associated with an increased risk of colon cancer^29^, and NLRP3 inflammasome activation is increased in human CRC tissues. This activation is associated with increased production of IL-1β and has also been associated with the EMT process and cancer progression^18,19^. In this context, *Alu* RNA trafficking via exosomes could be one of the stimuli that activates the NLRP3 inflammasome in CRC cells.

We demonstrated that exosomal *Alu* RNA promotes tumorigenesis by inducing NLRP3 inflammasome activation and EMT in CRC cell lines. Our findings are supported by an analysis of human CRC tumors, in which we previously found that *Alu* RNA expression is correlated with progression and metastasis^14^. Here, we observed that the accumulation *of Alu* RNA in CRC human tissues is associated with NLRP3 inflammasome priming. Although further studies are needed to elucidate the molecular mechanism underlying *Alu* RNA-induced inflammasome activation in CRC cells, these results strongly suggest that inflammasome activation occurs in vivo.

Interestingly, a recent study revealed that CRC cells express abundant repetitive elements, including long interspersed nuclear element-1 (LINE-1) retrotransposons, *Alu* RNAs and satellite repeats. These transposable elements have a viral-like cycle that can be therapeutically targeted with nucleoside reverse transcriptase inhibitors (NRTIs), and this approach prevented CRC progression in preclinical models^30^. Notably, we showed that NRTIs can inhibit *Alu* RNA-mediated signaling by inhibiting P2X7-mediated NLRP3 inflammasome activation, independent of reverse transcriptase inhibition^31^. In this context, our study confirms and reinforces the importance of *Alu* repetitive elements in CRC progression and provides another potential therapeutic strategy. Furthermore, it has recently been demonstrated that fluoxetine, which is a drug that is approved by the FDA for the treatment of clinical depression, can bind to and inhibit NLRP3 and, consequently, *Alu* RNA-dependent signaling^32^, thus increasing the overall survival of cancer patients treated with PD-1/L1 immunotherapy^33^; these results highlight the importance of NLRP3 signaling in cancer.

Exosomes have been shown to transfer their cargos from donor cells to recipient cells and to be responsible for cancer-induced vascular permeability, inflammation, and the formation of a premetastatic niche^34,35^. In this manuscript, we studied how exosomal *Alu* RNA affects CRC cell behavior, but the findings do not exclude the possibility that the transfer of these transcripts could affect other noncancer cells that are part of the tumor microenvironment, such as endothelial cells, inflammatory cells, and fibroblasts. Since inflammatory cells, such as macrophages, express high levels of NLRP3, this area of investigation will be particularly intriguing.

Recent studies have revealed an emerging role of exosomes from cancer patient serum as reliable markers for cancer diagnosis and prognosis assessment. Tumor microvesicles have been shown to contain retrotransposon elements, such as LINE-1 and *Alu* elements^15^. Here, we showed that in a preclinical tumor model, the abundance of *Alu* in exosomes from serum was associated with tumor progression and could be used as a marker for cancer diagnosis.

In conclusion, our findings reveal that *Alu* transcripts that are transferred via exosomes can positively affect colorectal cancer progression by activating the NLRP3 inflammasome, thus providing new insights into the important role of *Alu* sequences in cancer progression and providing new directions for the development of novel diagnostic and therapeutic strategies.

### Supplementary information

Supplementary Figures
