# Supplementary material for: Cancer-derived exosomal Alu RNA promotes colorectal cancer progression
Source: Exp Mol Med. 2024 Mar 14;56(3):700–10. doi: 10.1038/s12276-024-01166-6 (PMC10984964; doi:10.1038/s12276-024-01166-6)
Supplement: Supplementary file 1 — Supplementary Figures [file 12276_2024_1166_MOESM1_ESM.pdf]

Supplementary Fig. 1

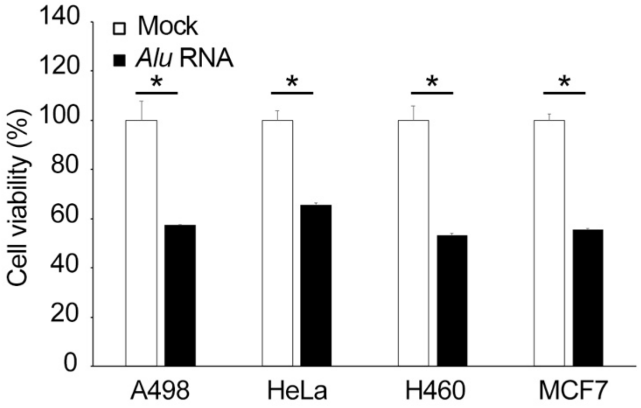

Alu RNA induces cell death in several cancer cell lines from different tissues: A498 (kidney), HeLa (cervix), H460 (lung), and MCF7 (breast), as monitored by MTT assay (n=3); *$p<0.05$. Error bars denote s.e.m.

Supplementary Fig. 2

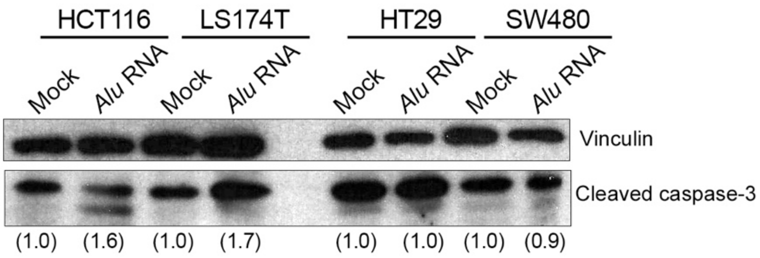

Western blot analysis for cleaved Caspase-3 (p17 and p19) of HCT116, LS174T, HT29, and SW480 cells after *Alu* RNA transfection. Densitometric values are normalized against Vinculin and are shown in parentheses.

Supplementary Fig. 3

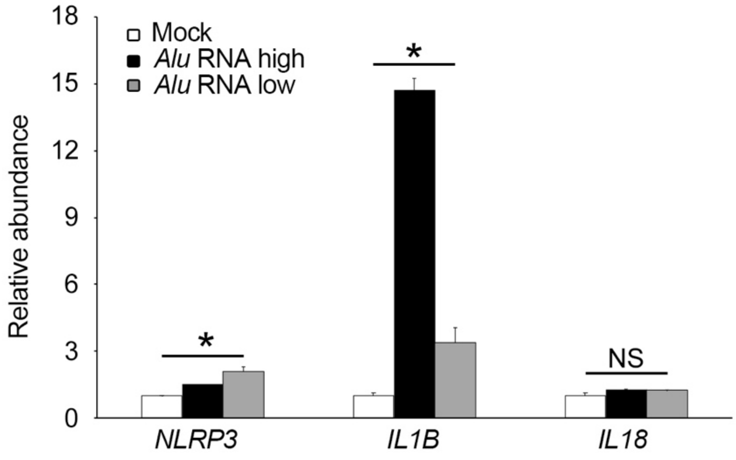

Transfection with high and low doses of *Alu* RNA induces the priming of the NLRP3 inflammasome by increasing the abundance of *NLRP3*, *IL-1B* mRNAs in HCT116 cells as evaluated by quantitative reverse transcriptase (qRT-PCR). No difference in *IL18* gene expression were observed, (n=3); *p<0.05. NS = not statistically significant. Error bars denote s.e.m.

Supplementary Fig. 4

a)

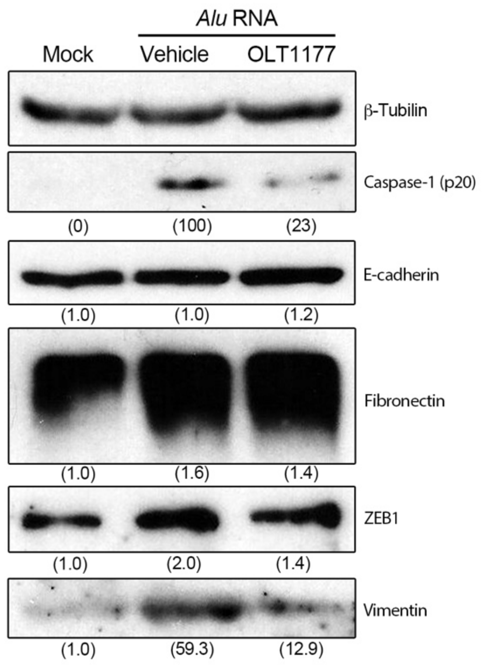

b)

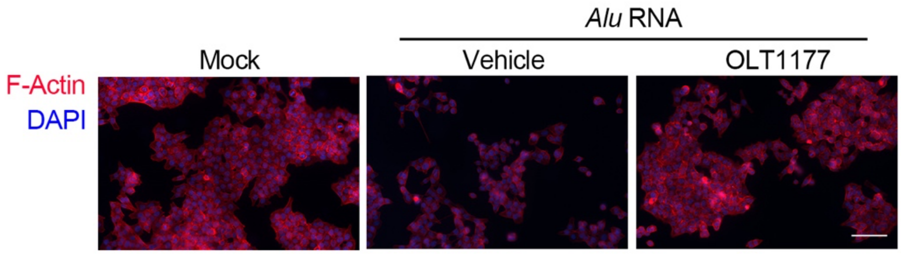

a) OLT1177 (100 μM) reduces *Alu* RNA-induced EMT in HCT116 cells, as determined by western blot analysis. *Alu* RNA (central lane) induces Caspase-1 activation as well as increasing Fibronectin, Vimentin and ZEB-1 compared to Mock (left lane). While the transfection of *Alu* RNA in HCT116 cells treated with OLT1177 rescues the *Alu*-induced EMT inhibiting *Alu*-induced Caspase-1 activation (right lane). No variation of E-cadherin are observed. Densitometric values are normalized against β-Tubulin and are shown in parentheses. **b)** Representative images of F-actin with rhodamine phalloidin (red). Nuclei are counterstained with 4',6-diamidino-2-phenylindole (DAPI, blue). Scale bar: 100 μm.

Supplementary Fig. 5

a)

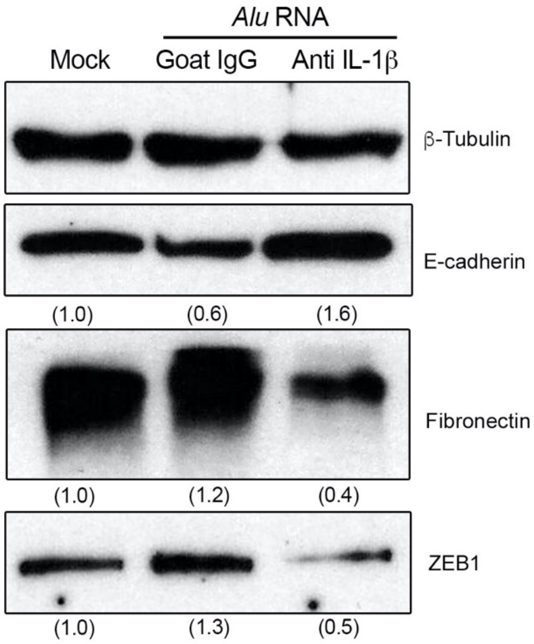

b)

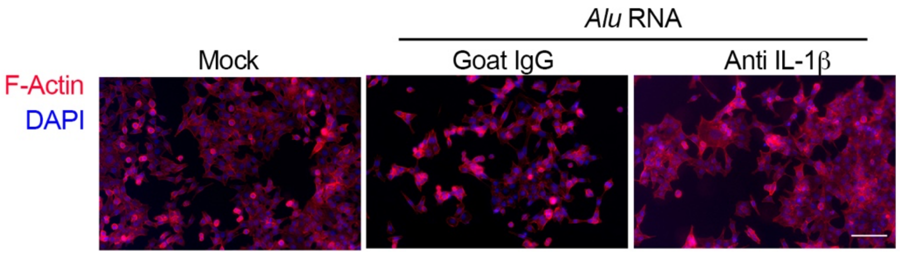

a) Anti IL-1β (500ng/ml) reduces *Alu* RNA-induced EMT in HCT116 cells, as determined by western blot analysis. *Alu* RNA (central lane) induces reduction of E-cadherin and increase of Fibronectin and ZEB-1 as compared to Mock (left lane). While the transfection of *Alu* RNA in HCT116 cells treated with Anti IL-1β rescues the *Alu*-induced EMT (right lane). Densitometric values are normalized against β-Tubulin and are shown in parentheses. **b)** Representative images of F-actin with rhodamine phalloidin (red). Nuclei are counterstained with DAPI, blue. Scale bar: 100 μm.

Supplementary Fig. 6

a)

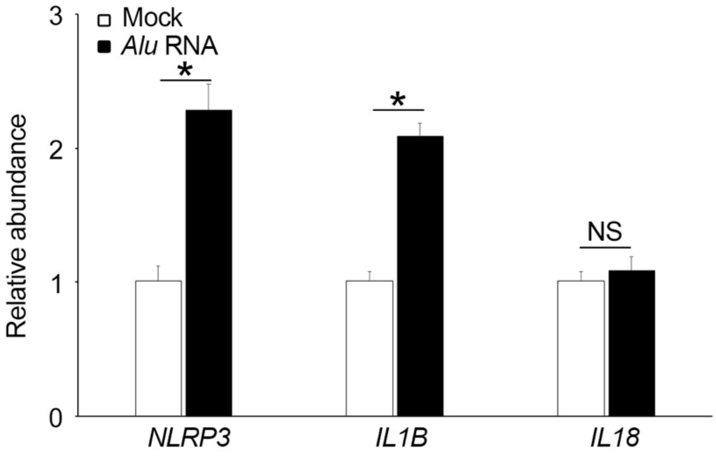

b)

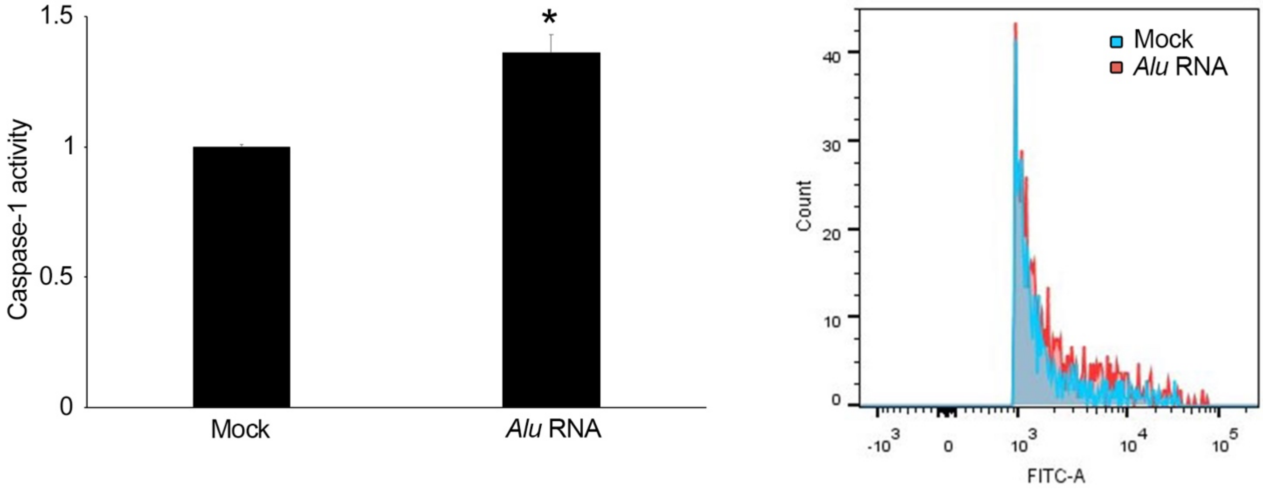

Alu RNA induces priming and activation of NLRP3 inflammasome in SW480 CRC cell line. **a**) *Alu* RNA induces the priming of the NLRP3 inflammasome by increasing the abundance of *NLRP3*, and *IL-1B* mRNAs in SW480 cells, as monitored by qRT-PCR. No difference in *IL18* gene expression were observed (n=3); *p<0.05. NS = not statistically significant. Error bars denote s.e.m. **b)** On the left, quantification of Caspase-1 activity was assessed by fluorometric assay on SW480 cells transfected with *Alu* RNA (n=3); *p<0.05. Error bars denote s.e.m. On the right, representative flow cytometry overlay plot showing increased fluorescence intensity of Caspase-1 activity in SW480 cells transfected with *Alu* RNA (in red) as compared with control cells (Mock, in light blue).

Supplementary Fig. 7

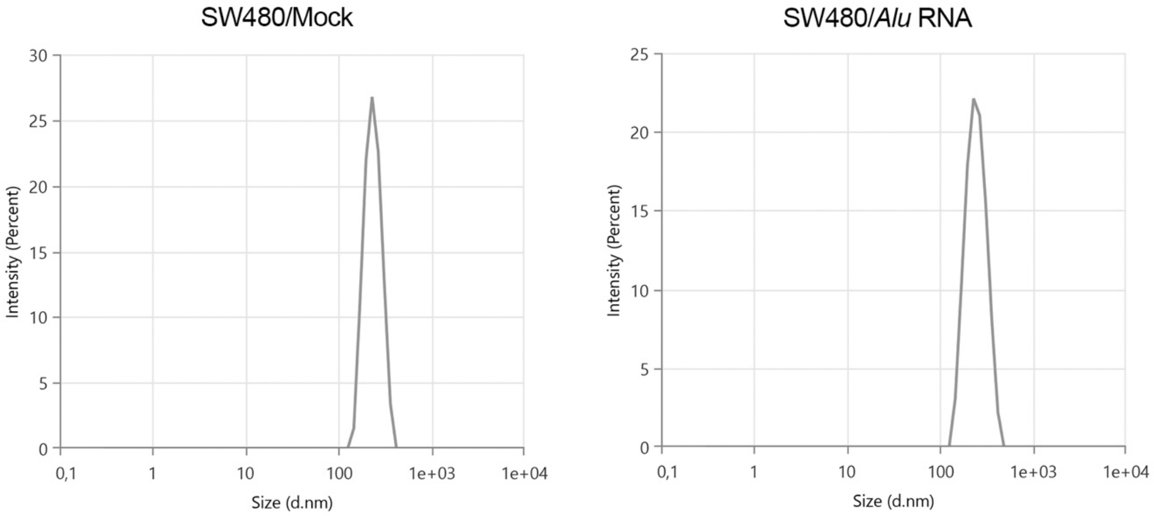

Representative Dynamic light scattering (DLS) results for the size distribution of exosomes purified from SW480 cells transfected with *Alu* RNA and with vehicle (Mock).

Supplementary Fig. 8

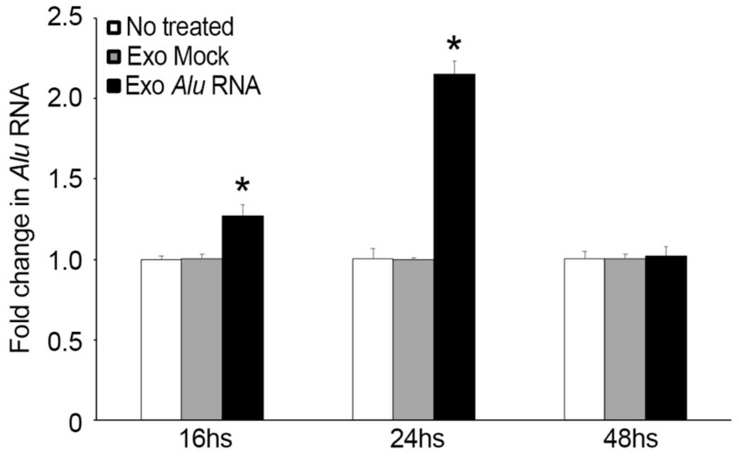

qRT-PCR shows *Alu* RNA abundance in SW480 cells treated with exosomes derived from *Alu*-transfected SW480 (Exo *Alu* RNA) after 16, 24 and 48 hours as compared with SW480 cells treated with control exosomes (Exo Mock) and untreated cells (n=3); *p<0.05. Error bars denote s.e.m.

Supplementary Fig. 9

a)

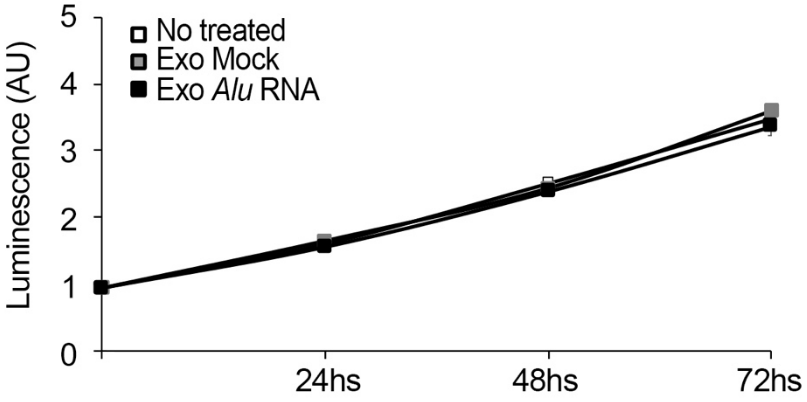

b)

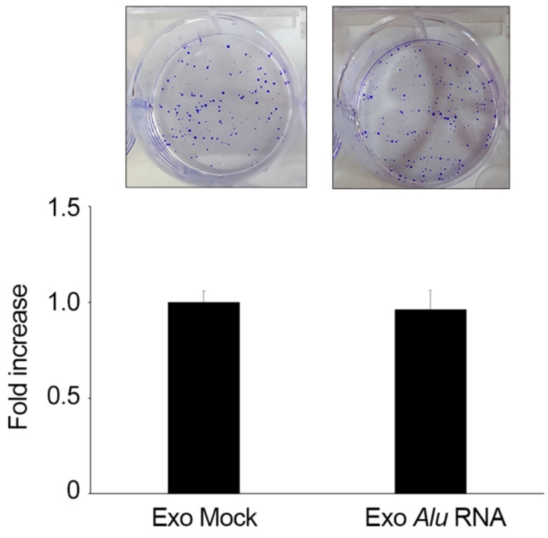

a) Exosomal-*Alu* RNA does not alter SW480 cell viability over time, as monitored by luminescent assay (n=3). **b)** Exosomal-*Alu* RNA does not alter the ability of SW480 to form colonies. Data are expressed as fold increase with the respect to SW480 treated with Exo Mock (n=3). Error bars denote s.e.m. Representative pictures of the two conditions are shown.

Supplementary Fig. 10

a)

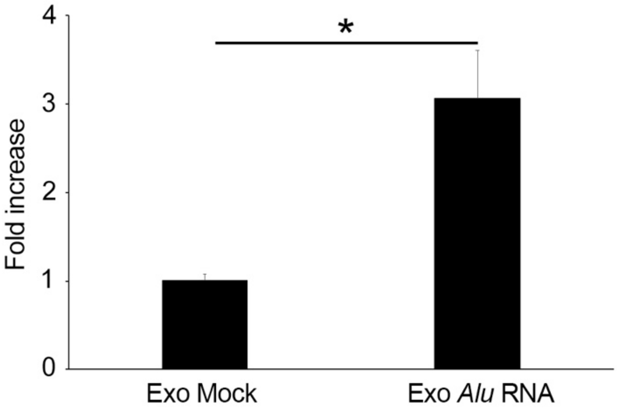

b)

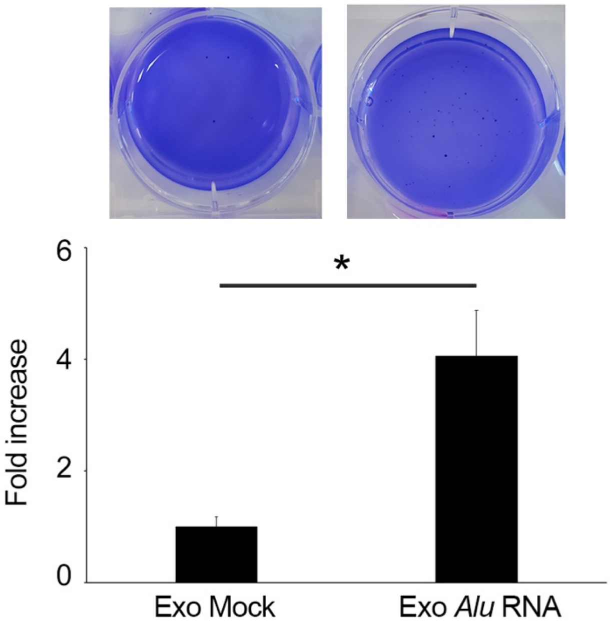

Cancer-derived exosomal-*Alu* RNA promotes tumorigenesis in SW480 CRC cell line. **a)** Exosomal-*Alu* RNA increases SW480 cell invasion. Data are expressed as fold increase with the respect to SW480 treated with Exo Mock (n=3); *p=0.002. Error bars denote s.e.m. **b)** Exosomal-*Alu* RNA increases SW480 ability to grow in anchorage-dependent manner. The bars indicate the average of colony numbers in each well (n=3); *p=0.005. Error bars denote s.e.m. Representative pictures of two conditions are shown.

Supplementary Fig. 11

a)

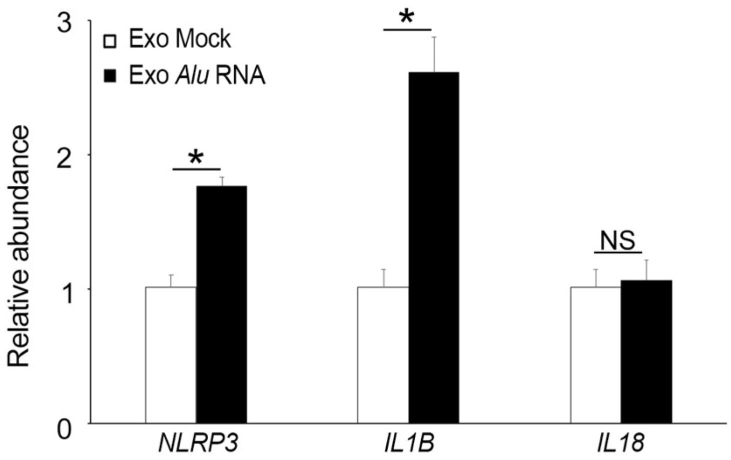

b)

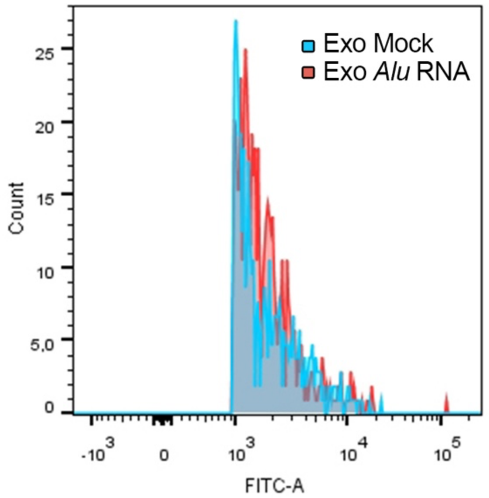

Exosomal-*Alu* RNA induces priming and activation of NLRP3 inflammasome in SW480 CRC cell line. **a)** Exosomal-*Alu* RNA induces the priming of the NLRP3 inflammasome by increasing the abundance of *NLRP3* and *IL-1B* mRNAs in SW480 cells, as evaluated by qRT-PCR (n=3); *p<0.05. No difference in *IL18* gene expression were observed. Error bars denote s.e.m. **b)** Representative flow cytometry overlay plot showing increased fluorescence intensity of Caspase-1 activity in SW480 cells treated with Exo *Alu* RNA (in red) as compared with cells treated with control exosomes (Exo Mock, in light blue).

Supplementary Fig. 12

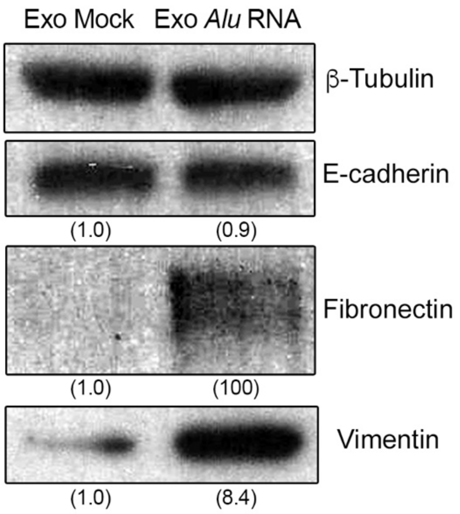

Exosomal-*Alu* RNA induces EMT in SW480 CRC cell line. Western blot analysis shows a decrease of E-cadherin, an increase of the mesenchymal marker (Fibronectin and Vimentin) in SW480 cells treated with Exo *Alu* RNA. Densitometric values are normalized against β-Tubulin and are shown in parentheses.

Supplementary Fig. 13

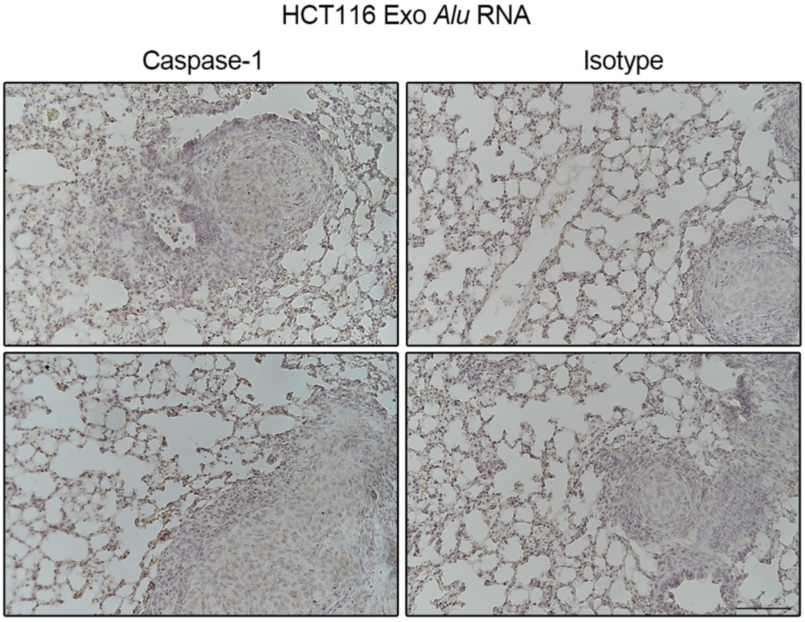

Representative pictures of anti-Cleaved Caspase-1 staining (left) and Isotype control antibody (right) of lung sections explanted from injected with HCT116 pre-incubated with exosomes enriched in *Alu* RNA are shown. Scale bar: 100 µm.
